# Does the antimicrobial-impregnated peripherally inserted central catheter decrease the CLABSI rate in neonates? Results from a retrospective cohort study

**DOI:** 10.3389/fped.2022.1012800

**Published:** 2022-11-24

**Authors:** Mohammad A. A. Bayoumi, Matheus F. P. T. van Rens, Prem Chandra, Alaa Masry, Sunitha D’Souza, Amr M. Khalil, Afaf Shadad, Safaa Alsayigh, Razan M. Masri, Sunitha Shyam, Fatima Alobaidan, Einas E. Elmalik

**Affiliations:** ^1^ Neonatal Intensive Care Unit (NICU), Women’s Wellness and Research Center (WWRC), Hamad Medical Corporation (HMC), Doha, Qatar; ^2^ Medical Research Center, Hamad Medical Corporation (HMC), Doha, Qatar; ^3^ Department of Medical Education, Hamad Medical Corporation (HMC), Doha, Qatar; ^4^ VERITADYNE Strategic Consulting Pvt. Ltd., Delhi, India

**Keywords:** vascular access, vascular access devices, peripherally inserted central catheter, antimicrobial-impregnated central venous catheters, central line-associated bloodstream infection, newborn, neonatal intensive care unit, neonate

## Abstract

**Background:**

The use of antimicrobial-impregnated peripherally inserted central catheters (PICCs) has been introduced in the last few years to neonatal units aiming to reduce central line-associated bloodstream infection (CLABSI).

**Methods:**

This retrospective observational study aimed to compare the CLABSI rates and other catheter-related parameters including the insertion success rates and catheter-related complications in the antimicrobial-impregnated and conventional (ordinary) PICCs in NICU between 2017 and 2020.

**Results:**

Our dedicated PICC team including physicians and nurses inserted 1,242 conventional (PremiCath and NutriLine) and 791 antimicrobial-impregnated PICCs (PremiStar) over the study period from 2017 to 2020. Of those 1,242 conventional PICCs, 1,171 (94.3%) were 1 Fr single lumen and only 71 (5.7%) were 2 Fr double lumen. The mean ± SD [median (IQR)] for the birth weight in all babies who had a PICC line was 1,343.3 ± 686.75 [1,200 (900, 1,500)] g, while the mean ± SD for the gestational age was 29.6 ± 4.03 [29 (27, 31)] weeks. The mean ± SD [median (IQR)] age at the time of insertion for all catheters was 9.3 ± 21.47 [2 (1, 9)] days, while the mean ± SD [median (IQR)] dwell time was 15.7 ± 14.03 [12 (8, 17)] days. The overall success rate of the PICC insertion is 1,815/2,033 (89.3%), while the first attempt success rate is 1,290/2,033 (63.5%). The mean ± SD [median (IQR)] gestational age, birth weight, age at catheter insertion, and catheter dwell time were 28.8 ± 3.24 [29, (26, 31)] weeks, 1,192.1 ± 410.3 [1,150, (900, 1,450)] g, 6.3 ± 10.85 [2, (1, 8)] days, and 17.73 ± 17.532 [13, (9, 18)] days in the antimicrobial-impregnated catheter compared with 30.1 ± 4.39 [29, (27, 32)] weeks (*P* < 0.001), 1,439.5 ± 800.8 [1,240, (920, 1,520)] g (*P* < 0.001), 11.1 ± 25.9 [1, (1, 9)] days (*P* < 0.001), and 14.30 ± 10.964 [12, (8, 17)] days (*P* < 0.001), respectively, in the conventional PICCs. The use of the antimicrobial-impregnated catheter was not associated with any significant reduction in the CLABSI rate (per 1,000 days dwell time), either the overall [*P* = 0.11, risk ratio (RR) (95% CI): 0.60 (0.32, 1.13)] or the yearly CLABSI rates.

**Conclusions:**

The use of miconazole and rifampicin-impregnated PICCs did not reduce the CLABSI rate in neonates compared with conventional PICCs. However, it has a higher overall rate of elective removal after completion of therapy and less extravasation/infiltration, occlusion, and phlebitis compared with the conventional PICCs. Further large RCTs are recommended to enrich the current paucity of evidence and to reduce the risk of bias. Neonatal PICCs impregnation by other antimicrobials is a recommendation for vascular access device manufacturers.

## Introduction

Since its first discovery in 1973, peripherally inserted central catheters (PICCs) have been progressively used in neonatal intensive care units (NICUs) (1). PICCs offer long-term venous access for neonates and are indicated for total parental nutrition (TPN), long-term IV medications and antibiotic therapy, and vesicant drug therapy (2, 3).

Central line-associated bloodstream infection (CLABSI) is the most common and serious complication related to PICC insertion in neonates. The causative organisms usually adhere and stick to the catheter and secrete a biologically coated film that protects them from systemically administered antimicrobials, allowing for prolonged microbial colonization (4–6). The organisms most frequently isolated from preterm babies in hospital-acquired infections are coagulase-negative Staphylococci, and other gram-positive cocci (*Staphylococcus aureus* and Enterococci), gram-negative bacilli (*E. coli*, *Pseudomonas aeruginosa*, and *Klebsiella pneumoniae*), and fungi (*Candida* pathogens) (7). CLABSI is associated with death, serious morbidities especially in very preterm babies, increased healthcare costs, prolonged length of hospital stay, and subsequent long-term adverse neurodevelopmental outcomes and lung diseases (8, 9).

The use of antimicrobial-impregnated central venous catheters (CVCs) has been recently recommended for patients at high risk of infection in addition to all other preventive measures (10, 11). The antimicrobial-impregnated catheter PremiStar; Vygon, Swindon, UK is miconazole and rifampicin-impregnated PICC, designed and manufactured aiming to reduce the incidence of CLABSI in newborns, by the combined synergistic effect of the two medications (12). Extruded polyurethane tubes are placed into a solution made of two anti-infective substances. By thermal activation, the combination of rifampicin and miconazole is chemically incorporated into the structure of the catheter. Miconazole is a systemic antifungal medication proven to be effective against systemic fungal infections. Rifampicin is a systemic antibacterial medication that has been investigated and tested in rifampicin and minocycline CVCs impregnation in children and adults (13–16). PremiStar was introduced to our neonatal unit in August 2016.

There is a limited number of studies comparing antimicrobial-impregnated vs. conventional PICCs in neonates that necessitated further research studies to compare both types of catheters (17). This retrospective observational study aimed to compare the CLABSI rates and other catheter-related parameters including the insertion success rates and catheter-related complications in the antimicrobial-impregnated vs. conventional PICCs in NICU from 2017 to 2020. The main objective is to assess the ability of the antimicrobial-impregnated PICCs in reducing the CLABSI rate in the NICU compared with the conventional PICCs using retrospectively collected data.

## Materials and methods

This is a retrospective observational study conducted in the NICU, Women's Wellness and Research Center (WWRC) at Hamad Medical Corporation (HMC), Doha, Qatar. The NICU in WWRC is a tertiary-level neonatal unit with 112 cots. The hospital has more than 18,000 deliveries per year and the NICU has more than 3,000 admissions per year.

In 2017, the PICC insertion team was developed and introduced to our NICU. Since then, the members are expanding and currently, the team includes 15 neonatologists, 1 neonatal nurse practitioner, and 7 NICU nurses. The central line simulation course is an accredited course that was designed by mobile pediatric simulation (MPS) and the neonatal simulation team based on the educational needs assessment. The aim was to train the team members and enhance their cognitive, technical and behavioral skills related to central line insertion. The participants were trained to insert PICC by MicroFlash insertion, the split steel needle insertion and the modified Seldinger technique (MST) (18–20). Since its launch, the PICC team has been working in collaboration with the neonatal specialized nursing (NSN) team. NSN members are highly trained NICU competent in IV line insertion. The NSN team's roles are the determination of the insertion eligibility criteria, VAD selection, line maintenance ensuring closed system care, blood samples collection, and insertion data entry in the specially designed online data collection system. In our day-to-day practice, there is no difference in the line maintenance quality, personnel, and frequency of all types of CVCs (21, 22).

In our NICU, we insert PICC for very low birth weight (VLBW) infants who are less than 1.5 kg, if intravenous fluids are needed for more than 5 days, if intravenous medications are needed for more than 7 days, the osmolality of the intravenous fluids exceeds 700 mOsmol/L and if the patient requires more than 3 peripheral intravenous catheters (PIVCs) insertions in 24 h. This is according to the locally developed protocol based on international guidelines (23).

Two types of conventional (ordinary) PICCs are available in our NICU (NutriLine 2 Fr; Vygon and PremiCath 1 Fr; Vygon). The PremiStar 1 Fr; Vygon, Swindon, UK is the only antimicrobial-impregnated PICC available in our unit. Based on our vascular access device (VAD) selection criteria, we insert PremiStar for extremely preterm infants less than 28 weeks gestation babies or in babies with suspected early or late-onset neonatal sepsis in the presence of risk factors. In big babies with older gestational ages or sick babies when a double lumen catheter is needed, NutriLine 2 Fr; 30 cm length; Vygon, was the right choice. For the rest of the babies who required PICC insertion, PremiCath 1 Fr; Vygon was used. The most frequently used veins for PICC insertions in the lower extremities were the great saphenous vein, the small saphenous vein, and the posterior tibial vein. The most frequently used veins for PICC insertions in the upper extremities were the antecubital vein, the cephalic vein, the basilic vein, and the ulnar vein. For all catheters, either split steel needle, MicroFlash over-the-needle technique, or MST was followed for the catheter insertion. A central venous catheter was considered to be successfully inserted when its tip was located either in the superior vena cava (SVC) or inferior vena cava (IVC) just outside the right atrium. As per our evidence-based guidelines for the insertion and management of PICC in neonates, two pricks are allowed for each PICC team member. After a total of three failed insertion attempts, the procedure was stopped. Exceptions are very limited with chronic patients who have difficult vascular access. Preventive removal refers to the unnecessary premature catheter withdrawal before completion of the IV therapy (21, 22, 24, 25).

The line maintenance and care bundles for preventing CLABSI in our unit include a fully sterile insertion bundle, a daily VAD assessment bundle, a bundle for change of the line dressing, and a catheter access bundle. The core bundle elements include but are not limited to applying maximal personal protective equipment (PPE), proper hand hygiene techniques, optimal catheter site, and device selection, a daily central line needs assessment, using a specialized central line trolley named Mayo Stand, performing the procedure time out, timing the duration of the procedure, using a central line insertion sterile closed kit, cleaning the line hub using chlorhexidine, applying a transparent semipermeable line dressing, using a needleless connector, using an alcohol-impregnated line port protector, applying two-person technique in each step all the time, using cyanoacrylate glue for catheter fixation and sealing of the insertion site, using diluted lipid emulsion solution as a lubricant to facilitate the guidewire removal, using the vein viewer imaging system for vascular assessment, using the microsite and the MST for the catheter insertion, early PICC insertion-early PICC removal approach, use of antimicrobial-impregnated catheters, speak-up campaigns to pick up any infection control breaches, and meticulously keeping a closed IV tubing system all the time. We have locally developed a mnemonic for neonatal vascular access pre-briefing, the “5Rights for Vascular Access” that is, choosing the Right vascular access device, for the Right patient, administering the Right therapy, in the Right vein, for the Right duration (24–28).

The Centers for Disease Control and Prevention (CDC) defines CLABSI as a laboratory-confirmed bloodstream infection not related to an infection at any other site, which develops within 48 h of central line insertion. The CLABSI rate is defined as the number of CLABSI infections per 1,000 central line days (29–31). These CDC definitions for CLABSI and CLABSI rate were followed in our unit.

An electronic web-based data registry was designed to collect the study variables and catheter-related parameters in both groups.

### Statistical analysis

The primary analysis in this observational research study was to evaluate and compare the CLABSI rates and other catheter-related parameters including the insertion success rates and catheter-related complications between the antimicrobial-impregnated and conventional PICCs in NICU. Descriptive statistics were used to summarize the sample characteristics using mean and standard deviation (SD) for normally distributed data and median and interquartile range (IQR) for skewed data. Qualitative data were summarized using frequencies and percentages. Associations between two or more categorical variables were compared using Chi-square (*χ*^2^) test or Fisher Exact test as applicable. Quantitative data between the two independent groups (e.g., antimicrobial-impregnated and conventional catheters; CLABSI vs. non-CLABSI, etc.) were analyzed using a *t*-test or nonparametric Mann–Whitney *U*-test as appropriate.

Logistic regression analysis was used to examine and evaluate the potential confounders and covariates (as shown in Tables 4, 5) associated with CLABSI. For multivariate logistic regression models, predictor variables were included in consideration with both statistical and clinical significance and relevance. The results of logistic regression analysis were presented as odds ratios (ORs) with corresponding 95% confidence intervals (CIs). Subsequently, we used the receiver operating characteristic curve (ROC) to evaluate the discriminative ability (predictive accuracy of the developed logistic regression model) of potentially significant predictors associated with CLABSI. The linear relationship between two continuous variables was assessed using Pearson's or Spearman's correlation coefficients. Two-tailed *P-*values <0.05 were considered statistically significant. It was written as numbers and if it is very low, i.e., less than 0.001, it was put as <0.001. All statistical analyses were performed using statistical packages SPSS version 28.0 (Armonk, NY: IBM Corp) and Epi-info (Centers for Disease Control and Prevention, Atlanta, GA) software.

## Results

Our dedicated PICC team including physicians and nurses inserted 1,242 conventional (PremiCath and NutriLine) and 791 antimicrobial-impregnated PICCs (PremiStar) over the study period from 2017 to 2020. Of those 1,242 conventional PICCs, 1,171 (94.3%) were 1 Fr single lumen and only 71 (5.7%) were 2 Fr double lumen. Table 1 shows the mean ± SD [median (IQR)] for the birth weight, gestational age, age at the time of insertion, and catheter dwell time for the whole study population.

**Table 1:** Demographics of the study sample.

Variables	Mean ± SD [median (IQR)]
Gestational age at birth (weeks)	29.6 ± 4.03 [29 (27, 31)]
<28 weeks	655 (32.2%)
28 to <32 weeks	889 (43.7%)
32 to 36 weeks	311 (15.3%)
>36 weeks	178 (8.8%)
Age at insertion (days)	9.3 ± 21.47 [2 (1, 9)]
Birth weight (g)	1,343.3 ± 686.75 [1,200 (900, 1,500)]
<1,000 g	663 (32.6%)
1,000 to <1,500 g	860 (42.3%)
1,500 to 2,500 g	340 (16.7%)
>2,500 g	170 (8.4%)
Dwell time (days)	15.7 ± 14.03 [12 (8, 17)]

Median and interquartile ranges (IQRs) were used for skewed data.

All the percentage values were computed using nonmissing values.

**Figure 1:**
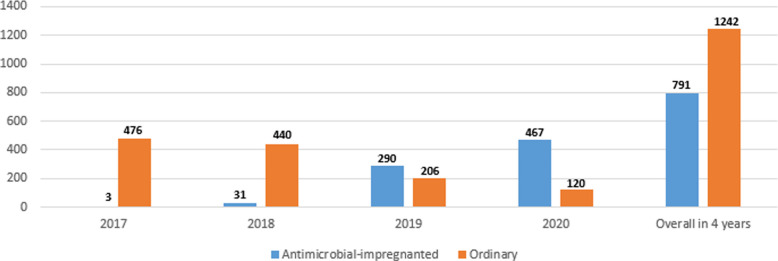
Numbers of the two types of catheters across the 4 years.

**Figure 2:**
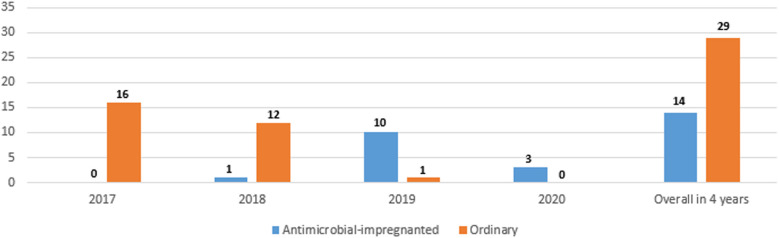
Number of CLABSI cases between the two types of catheters across the 4 years.

**Figure 3:**
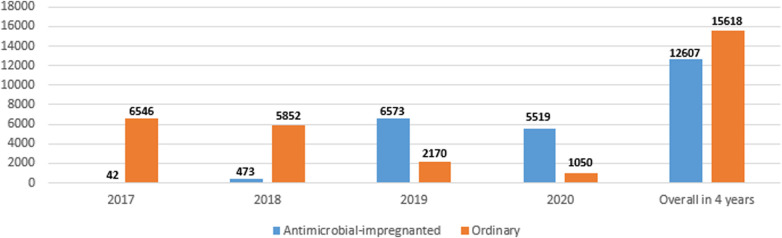
Dwell time in days between the two types of catheters across the 4 years.

Figure 1 shows the numbers of the two types of catheters across the 4 years showing a gradual increase in the use of antimicrobial-impregnated catheters. The main indication for PICC line insertion in our study was the need for long-term IV fluid therapy 1,815/2,033 (89.3%) and the most commonly used catheter was the single lumen conventional PICC 1,171/2,033 (57.6%) followed by the single lumen antimicrobial-impregnated PICC 791/2,033 (38.9%). The overall success rate of the PICC insertion is 1,815/2,033 (89.3%), while the first attempt success rate is 1,290/2,033 (63.5%). The main reason for the central venous catheter removal in our study was the elective removal after completion of therapy 1,449/2,033 (79.8%). We had 43/2,033 (2.1%) CLABSI cases in our study sample (Table 2). Figure 2 shows the number of CLABSI cases between the two types of catheters across the 4 years and Figure 3 shows the dwell time in days between the two types of catheters across the 4 years.

**Table 2:** Clinical variables of the study sample.

Variables	(*n* = 2,033) (%)
Gender	
Male	1,106 (54.4%)
Female	927 (45.6%)
Reason for insertion	
Long-term IV fluid therapy	1,815 (89.3%)
Long-term IV medication therapy	54 (2.7%)
Hypoglycemia	58 (2.9%)
Difficult IV insertion	106 (5.2%)
Catheter type	
Antimicrobial-impregnated	791 (38.9%)
Conventional	1,242 (61.1%)
Number of catheter lumens	
Single lumen antimicrobial-impregnated	791 (38.9%)
Single lumen conventional	1,171 (57.6%)
Double lumen conventional	71 (3.5%)
Insertion technique	
MST	1,103 (70.4%)
MicroFlash	356 (22.7%)
Split steel needle	107 (6.8%)
No. of attempts/pricks	
1	1,290 (63.5%)
2	439 (21.6%)
3	253 (12.4%)
≥4	51 (2.5%)
Site of insertion	
Lower extremities	1,539 (75.7%)
Upper extremities	493 (24.3%)
Side of the body	
Right	1,325 (65.2%)
Left	708 (34.8%)
Successful insertion	
No	218 (10.7%)
Yes	1,815 (89.3%)
Glue applied	
No	951 (52.3%)
Yes	866 (47.7%)
Type of preflush	
Normal saline	1,554 (76.5%)
Lipid emulsion	478 (23.5%)
Resistance during the guidewire removal	
No	602 (85.3%)
Yes	104 (14.7%)
Reason for removal	
Elective removal	1,449 (79.8%)
Accidental removal	8 (0.4%)
Broken catheter	14 (0.8%)
Leaking	28 (1.5%)
Extravasation/infiltration	43 (2.4%)
Malposition	22 (1.2%)
Occlusion	42 (2.3%)
Phlebitis	55 (3.0%)
CLABSI	43 (2.4%)
Preventive	78 (4.3%)
Death	34 (1.9%)
CLABSI	
No	1,990 (97.9%)
Yes	43 (2.1%)

CLABSI, central line-associated bloodstream infection; MST, modified Seldinger technique.

All the percentage values were computed using nonmissing values.

Table 3 shows the mean ± SD [median (IQR)] gestational age, birth weight, age at catheter insertion, and catheter dwell time in the antimicrobial-impregnated catheter compared with the conventional PICCs.

**Table 3:** Association between catheter types and other parameters.

Parameters	Antimicrobial-impregnated catheters, *n* (%) (*N* = 791)	Conventional catheters, *n* (%) (*N* = 1,242)	*P*-value
Gestational age at birth (weeks)	28.8 ± 3.24 (median 29, IQR 26, 31)	30.1 ± 4.39 (median 29, IQR 27, 32)	<0.001
<28 weeks	315 (39.8%)	340 (27.4%)	<0.001
28 to <32 weeks	342 (43.2%)	547 (44.0%)	
32 to 36 weeks	120 (15.2%)	191 (15.4%)	
>36 weeks	14 (1.8%)	164 (13.2%)	
Age at insertion (days)	6.3 ± 10.85 (median 2, IQR 1, 8)	11.1 ± 25.9 (median 1, IQR 1, 9)	<0.001
Birth weight (g)	1,192.1 ± 410.3 (median 1,150, IQR 900, 1,450)	1,439.5 ± 800.8 (median 1,240, IQR 920, 1,520)	<0.001
<1,000 g	282 (35.7%)	381 (30.7%)	<0.001
1,000 to <1,500 g	339 (42.9%)	521 (41.9%)	
1,500 to 2,500 g	158 (20.0%)	182 (14.7%)	
>2,500 g	12 (1.5%)	158 (12.7%)	
Dwell time (days)	17.73 ± 17.532 (median 13, IQR 9, 18)	14.30 ± 10.964 (median 12, IQR 8, 17)	<0.001
Gender			
Male	438 (55.4%)	668 (53.8%)	0.483
Female	353 (44.6%)	574 (46.2%)	
Reason for insertion			
Long-term IV fluid therapy	715 (90.4%)	1,100 (88.6%)	0.027
Long-term IV medication therapy	27 (3.4%)	27 (2.2%)	
Hypoglycemia	15 (1.9%)	43 (3.5%)	
Difficult IV insertion	34 (4.3%)	72 (5.8%)	
Number of catheter lumens			
Single lumen	791 (100.0%)	1,171 (94.3%)	<0.001
Double lumen	0 (0.0%)	71 (5.7%)	
Insertion technique			
MST	694 (88.1%)	409 (52.6%)	<0.001
MicroFlash	53 (6.7%)	303 (38.9%)	
Split steel needle	41 (5.2%)	66 (8.5%)	
No. of attempts/pricks			
1	523 (66.1%)	767 (61.8%)	0.021
2	174 (22.0%)	265 (21.3%)	
3	78 (9.9%)	175 (14.1%)	
≥4	16 (2.0%)	35 (2.8%)	
Site of insertion			
Lower extremities	679 (85.8%)	860 (69.3%)	<0.001
Upper extremities	112 (14.2%)	381 (30.7%)	
Side of the body			
Right	566 (71.6%)	759 (61.1%)	<0.001
Left	225 (28.4%)	483 (38.9%)	
Successful insertion			
No	72 (9.1%)	146 (11.8%)	0.059
Yes	719 (90.9%)	1,096 (88.2%)	
Glue applied			
No	84 (11.7%)	867 (79.0%)	<0.001
Yes	635 (88.3%)	231 (21.0%)	
Type of preflush			
Normal saline	370 (46.8%)	1,184 (95.4%)	<0.001
Lipid emulsion	421 (53.2%)	57 (4.6%)	
Resistance during the guidewire removal			
No	487 (86.7%)	115 (79.9%)	0.040
Yes	75 (13.3%)	29 (20.1%)	
Reason for removal			
Elective removal	637 (88.6%)	812 (74.0%)	<0.001
Accidental removal	2 (0.3%)	6 (0.5%)	0.398
Broken catheter	6 (0.8%)	8 (0.7%)	0.802
Leaking	7 (1%)	21 (1.9%)	0.112
Extravasation/infiltration	3 (0.4%)	40 (3.6%)	<0.001
Malposition	7 (1%)	15 (1.4%)	0.453
Occlusion	10 (1.4%)	32 (2.9%)	0.034
Phlebitis	6 (0.8%)	49 (4.5%)	<0.001
CLABSI	14 (1.9%)	29 (2.6%)	0.340
Preventive	21 (2.9%)	57 (5.2%)	0.019
Death	6 (0.8%)	28 (2.6%)	0.008
CLABSI			
No	777 (98.2%)	1,213 (97.7%)	0.388
Yes	14 (1.8%)	29 (2.3%)	
Group of the CLABSI causative organism			
Gram-positive organism	4 (28.6%)	8 (27.6%)	0.946
Gram-negative organism	10 (71.4%)	21 (72.4%)	
The subgroup of the CLABSI causative organisms			
Gram-positive cocci	4 (28.6%)	7 (24.1%)	0.757
Gram-positive bacilli	0 (0%)	1 (3.4%)	
Gram-negative bacilli	10 (71.4%)	21 (72.4%)	

CLABSI, central line-associated bloodstream infection; MST, modified Seldinger technique; IQR, interquartile range.

Yates corrected Chi-square test was applied in case of small cell frequencies (50% or more cells have expected frequencies <5), whereas the quantitative outcome measures were compared using a *t*-test or nonparametric Mann–Whitney *U*-test as appropriate.

All the percentage values were computed using nonmissing values.

The majority of our PICCs were inserted in the lower extremities 1,539/2,033 (75.7%). The veins used for central line insertion in our study population are great saphenous 1,536 (75.6%), antecubital 364 (17.9%), cephalic 61 (3.0%), basilic 41 (2.0%), brachial 18 (0.9%), axillary 5 (0.2%), metacarpal 2 (0.1%), popliteal 2 (0.1%), temporal 2 (0.1%), and femoral 1 (0%) veins.

Of the 43 organisms causing CLABSI, 31 (72%) were gram-negative while the remaining 12 (28%) were gram-positive organisms. Of those gram-positive organisms, 1 (8.3%) was gram-positive bacilli, 9 (75%) were gram-positive cocci in clusters, and 2 (16.7%) were gram-positive cocci in pairs/chains. All the gram-negative organisms were gram-negative bacilli.

**Figure 4:**
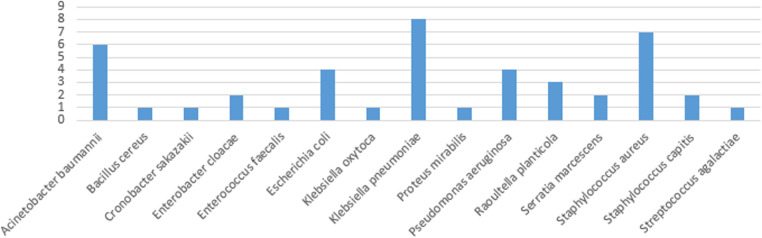
CLABSI-causative organisms in both types of catheters across the 4 years.

The use of the antimicrobial-impregnated catheter was not associated with any significant reduction in the CLABSI rate (per 1,000 days dwell time), either the overall [*P* = 0.11, risk ratio (RR) (95% CI): 0.60 (0.32, 1.13)] or the yearly CLABSI rates (Table 4). The CLABSI-causative organisms in both types of catheters in the 4 years are shown in Figure 4.

**Table 4:** CLABSI rates (per 1,000 days dwell time) between the two types of catheters across the 4 years.

Year	CLABSI rate in antimicrobial-impregnated catheters	CLABSI rate in conventional catheters	Risk ratio (RR) (95% CI)	*P*-value
2017	0	2.44	NA	0.843
2018	2.11	2.05	1.03 (0.13, 7.91)	0.977
2019	1.52	0.46	3.30 (0.42, 25.77)	0.227
2020	0.54	0	NA	0.593
Overall in the 4 years	1.11	1.86	0.60 (0.32, 1.13)	0.11

CLABSI, central line-associated bloodstream infection; CI, confidence interval; NA, not applicable.

CLABSI rate is the number of CLABSI cases per 1,000 days dwell time.

Univariate and multivariate logistic regression analysis was performed to test for the potential factors, predictors and possible association with the dichotomous outcome variable (CLABSI and non-CLABSI). Results of the univariate analysis indicated that gestational age at birth, birth weight, age at insertion, insertion techniques, cyanoacrylate glue application, and type of preflush had a significant association with CLABSI. The mean gestational age at birth for those who developed CLABSI was 26.58 ± 3.83 vs. 29.64 ± 4.01 weeks for those who did not, unadjusted odds ratio (95% CI) 0.75 (0.67, 0.85), *P* < 0.001. The mean birth weight for those who had CLABSI was 1,014.05 ± 743.06 vs. 1,350.36 ± 683.93 g for those who did not, unadjusted odds ratio (95% CI) 0.98 (0.98, 0.99), *P* = 0.001. The mean age at the central line insertion for those who developed CLABSI was 11.56 ± 18.64 vs. 9.23 ± 21.53 days for those who did not, unadjusted odds ratio (95% CI) 1.01 (0.99, 1.02), *P* = 0.084. The mean dwells time for those who had CLABSI was 20.49 ± 16.64 vs. 15.54 ± 13.94 days for those who did not, unadjusted odds ratio (95% CI) of 1.02 (1.01, 1.03), *P* = 0.036. The insertion technique most associated with CLABSI was the use of the split needle 7 (6.5%), unadjusted odds ratio (95% CI) 7.65 (2.85, 20.54), *P* < 0.001 compared with the MicroFlash 10 (2.8%), unadjusted odds ratio (95% CI) 3.16 (1.30, 7.65), *P* = 0.011 and MST which had the least CLABSI 10 (0.9%) (Table 5).

**Table 5:** Factors associated with CLABSI: univariate logistic regression analysis.

Variables	CLABSI (Yes), *n* (%) *N* = 43	Unadjusted odds ratio (95% CI)	*P*-value
Catheter type			
Antimicrobial-impregnated	14 (1.8%)	1.0 (reference)	
Conventional	29 (2.3%)	1.33 (0.69, 2.53)	0.390
Gestational age at birth			
<28 weeks	33 (5.0%)	1.0 (reference)	
28 to <32 weeks	6 (0.7%)	0.13 (0.05, 0.31)	<0.001
32 to 36 weeks	2 (0.6%)	0.12 (0.03, 0.51)	0.004
>36 weeks	2 (1.1%)	0.21 (0.05, 0.90)	0.036
Birth weight (g)			
<1,000 g	30 (4.5%)	1.0 (reference)	
1,000 to <1,500 g	9 (1.0%)	0.22 (0.11, 0.47)	<0.001
1,500 to 2,500 g	2 (0.6%)	0.13 (0.03, 0.53)	0.005
>2,500 g	2 (1.2%)	0.25 (0.06, 1.06)	0.060
Age at insertion (days)			
≤5 days	19 (1.4%)	1.0 (reference)	
5–10 days	16 (3.9%)	2.83 (1.44, 5.55)	0.003
>10 days	8 (2.8%)	1.97 (0.85, 4.53)	0.113
Dwell time (days)			
≤14 days	23 (2.0%)	1.0 (reference)	
>14 days	20 (3.2%)	1.64 (0.89, 3.01)	0.112
Gender			
Male	24 (2.2%)	1.0 (reference)	
Female	19 (2.0%)	0.94 (0.51, 1.73)	0.851
Reason for insertion			
Long-term IV fluid therapy	41 (2.3%)	1.0 (reference)	
Long-term IV medication therapy	2 (3.7%)	1.66 (0.39, 7.07)	0.490
Number of catheter lumens			
Single lumen antimicrobial-impregnated	14 (1.8%)	1.0 (reference)	
Single lumen conventional	29 (2.5%)	1.41 (0.74, 2.68)	0.297
Insertion technique			
MST	10 (0.9%)	1.0 (reference)	
MicroFlash	10 (2.8%)	3.16 (1.30, 7.66)	0.011
Split steel needle	7 (6.5%)	7.65 (2.85, 20.54)	<0.001
No. of attempts/pricks			
1	26 (2.0%)	1.0 (reference)	
2	14 (3.2%)	1.60 (0.83, 3.09)	0.161
3	3 (1.2%)	0.58 (0.18, 1.94)	0.380
Site of insertion			
Lower extremities	28 (1.8%)	1.0 (reference)	
Upper extremities	15 (3.0%)	1.69 (0.90, 3.26)	0.104
Side of the body			
Right	24 (1.8%)	1.0 (reference)	
Left	19 (2.7%)	1.50 (0.81, 2.75)	0.196
Glue applied			
No	30 (3.2%)	1.0 (reference)	
Yes	13 (1.5%)	0.47 (0.24, 0.90)	0.024
Type of preflush			
Normal saline	39 (2.5%)	1.0 (reference)	
Lipid emulsion	4 (0.8%)	0.33 (0.12, 0.92)	0.035

CI, confidence interval; CLABSI, central line-associated bloodstream infection; MST, modified Seldinger technique.

Outcome variable: the non-CLABSI group was considered the reference group.

All the percentage values were computed using nonmissing values.

**Figure 5:**
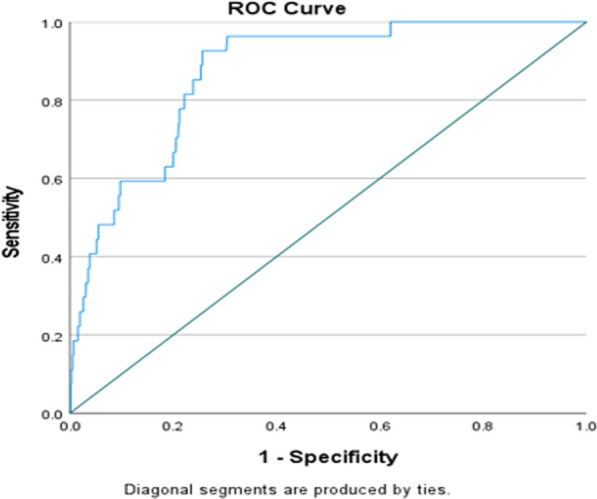
Area under the curve (AUC) value: 0.871 (95% CI, 0.82, 0.92).

The multivariate logistic regression analysis showed that gestational age at birth (adjusted OR 0.19, 95% CI, 0.07, 0.51, *P* < 0.001, when compared groups gestational age 28 to <32 weeks with reference group <28 weeks), number of attempts/pricks (adjusted OR 2.68, 95% CI, 1.19, 6.01, *P* = 0.017, when compared the number of attempts/pricks 2 with 1), and insertion techniques (adjusted OR 3.39, 95% CI, 1.37, 8.37, *P* = 0.008, when compared MicroFlash with reference group MST and adjusted OR 5.34, 95% CI, 1.91, 14.89, *P* = 0.001, when compared split steel needle with reference group MST) were associated with significantly more CLABSI after adjusting for other predictors and factors (Table 6). None of the clinical variables or catheter-related parameters was significantly associated with any gram stain type of the CLABSI-causative organisms in both groups. The discriminative ability of the significant predictors (observed in multivariate analysis) in predicting CLABSI was found to be good with an area under the ROC curve value of 0.871 (95% CI, 0.82, 0.92), which indicates that this developed regression model demonstrated an excellent fit (Figure 5).

**Table 6:** Predictors associated with CLABSI: multivariate logistic regression analysis.

Predictors	Adjusted odds ratio (OR)	95% CI for adjusted OR	*P*-value
Gestational age at birth			
<28 weeks	1.0 (reference)		
28 to <32 weeks	0.19	0.07, 0.51	<0.001
32 to 36 weeks	0.23	0.05, 1.01	0.052
>36 weeks	–	–	–
No. of attempts/pricks			
1	1.0 (reference)		
2	2.68	1.19, 6.01	0.017
3	0.35	0.04, 2.78	0.321
Insertion technique			
MST	1.0 (reference)		
MicroFlash	3.39	1.37, 8.37	0.008
Split steel needle	5.34	1.91, 14.89	0.001

CI, confidence interval; CLABSI, central line-associated bloodstream infection; MST, modified Seldinger technique.

Outcome variable: the non-CLABSI group was considered the reference group.

All the percentage values were computed using nonmissing values.

## Discussion

The neonatal antimicrobial-impregnated catheter was introduced to the market in December 2012 after getting the appropriate approvals and certification under the Conformité Européenne process (certificate number Z/12/02895) to reduce the rate and severity of CLABSI in NICUs. It has been used in many countries including Germany, Italy, and the UK. Despite the overall gradual decrease in the CLABSI rate in our NICU from 12.18 per 1,000 days dwell time in 2010 to 2.14 per 1,000 days dwell time in 2019, this improvement remained unsteady and showed a lack of sustainability (21, 22, 24). In 2016, the antimicrobial-impregnated PICC was introduced to the unit to help in reducing the CLABSI rate aiming for Zero-CLABSI.

From our study, no statistically significant differences or reductions in CLABSI rates were observed in neonates with the miconazole and rifampicin-impregnated catheters compared with those with the conventional PICCs. This applies to both the yearly and the overall CLABSI rates. In 2018, 2019, and 2020, the CLABSI rates were even higher in the antimicrobial-impregnated catheters compared with the conventional catheters; however, this difference was not statistically significant. This is related to the number of catheters in each category per year as displayed in Figure 1. The PREVAIL trial in neonates reported similar findings to ours with no evidence to support the use of the miconazole and rifampicin-impregnated catheters over the conventional catheter (12). The CATCH trial in children also reported no significant difference or superiority of the antimicrobial-impregnated catheters over the conventional ones (13). A meta-analysis to investigate the efficacy of the antimicrobial-impregnated catheters for the prevention of CLABSI in the neonatal and pediatric populations had also similar findings to ours and the PREVAIL trial (14). Furthermore, the antimicrobial-impregnated catheter; PremiStar is more expensive (QAR 720) compared with the conventional ones; PremiCath (QAR 305) and NutriLine (QAR 335). A randomized controlled trial was conducted in the UK to investigate the cost-effectiveness of different strategies to prevent late-onset sepsis in preterms and concluded that the antimicrobial-impregnated catheters are not likely to be cost-effective (32, 33). On the other hand, in a small trial available in the Cochrane database involving 98 patients, the authors found that silver zeolite-impregnated umbilical venous catheters (UVCs) significantly reduced the incidence of catheter-related bloodstream infections in preterms (34). A systematic review and network meta-analysis of the adult population demonstrated that antimicrobial-impregnated CVCs significantly reduced the CLABSI rate compared with standard catheters (15).

The early insertion, use of glue, MST insertion technique, single lumen catheters, a small number of attempts, and the use of lipid emulsion to facilitate the guidewire removal are all significantly higher in the miconazole and rifampicin-impregnated catheter and are all supposed to reduce the CLABSI rate (25, 35, 36). On the other hand, the birth weight and the gestational age were significantly lower in the miconazole and rifampicin-impregnated catheter group together with the longer dwell time compared with the conventional PICC. These factors are known to contribute to a higher CLABSI rate (37, 38). The multivariate logistic regression analysis of our results showed that the gestational age, the number of attempts, and the insertion technique are the predictors associated with CLABSI. However, the net result from our study is that the use of the miconazole and rifampicin-impregnated catheter did not improve the CLABSI rate compared with the conventional PICCs.

Results from our study show that the use of antimicrobial-impregnated catheters is not without benefits. Compared with conventional catheters, it has an overall elective removal rate of 637/791 (88.6%) compared with 812/1,242 (74.0%) in conventional catheters (*P* < 0.001). Also, the rate of extravasation/infiltration, occlusion, phlebitis, and preventive removals were all less in the antimicrobial-impregnated catheters (0.4%, 1.4%, 0.8%, and 2.9%) compared with the conventional ones (3.6% *P* < 0.001, 2.9% *P* = 0.034, 4.5% *P* = 0.001, and 5.2% *P* = 0.019), respectively. This can not be explained by the smaller caliber in the antimicrobial-impregnated catheters as most conventional PICC 1,171/1,242 (94.3%) has the same caliber. Preventive removals are related to the PICC team members who removed the line prematurely without a clear indication but extravasation/infiltration, occlusion, and phlebitis are related to the catheter itself. These complications have considerable clinical and healthcare cost impacts on neonates in NICU.

Materials used in central venous catheter impregnation in adults and pediatric populations available in the literature include antibiotics, such as vancomycin, teicoplanin, 5-fluorouracil, benzalkonium chloride, minocycline, minocycline/rifampicin, miconazole/rifampicin, and antiseptics; such as Oligon Vantex silver or silver, silver zeolite (AgION), chlorhexidine/silver sulfadiazine (15). To the best of our knowledge, the only type of neonatal antimicrobial-impregnated PICC is the one included in our study, which is impregnated with miconazole and rifampicin. These antimicrobials might not be fully and equally effective against the different CLABSI-causative organisms displayed in Figure 4. The emergence of resistant strains of the known CLABSI-causative organisms and the growth of new rare strains of bacteria should also be put into consideration. Those could be the reasons for our main study result stating the antimicrobial-impregnated catheters are not superior to the conventional ones in reducing the CLABSI rate. We encourage the manufacturers and vascular access industrial bodies to try the impregnation of neonatal catheters with other materials based on the types of neonatal CLABSI-causative organisms and their microbiome signatures (39).

The main limitation of our study is the retrospective and single-centered study design. However, the large sample size of 2,033 neonates increases its validity, power, and generalizability. Also, the two groups of neonates analyzed are quite different in terms of gestational age and weight. As many confounders might still exist, well-designed large-sample pragmatic randomized controlled trials (RCTs) are urgently indicated. Blinding both the caregivers and the study team is recommended to avoid introducing bias.

Reducing CLABSI is not dependent upon a single intervention. It is multifactorial and needs multidisciplinary team collaboration with continuous quality improvement projects. It remains a challenge to achieve and maintain zero-CLABSI in NICU.

## Conclusions

The use of miconazole and rifampicin-impregnated PICCs did not reduce the CLABSI rate in neonates compared with conventional PICCs. However, it has a higher overall rate of elective removal after completion of therapy and less extravasation/infiltration, occlusion, and phlebitis compared with the conventional PICCs. Further large RCTs are recommended to enrich the current paucity of evidence and to reduce the risk of bias. Neonatal PICCs impregnation by other antimicrobials is a recommendation for VAD manufacturers.

## Data Availability

The datasets generated for this study are available on reasonable request to the corresponding author. Data requests should be made to Dr. Mohammad A. A. Bayoumi by email to moh.abdelwahab@hotmail.com.
